# Overall Health Status of Patients with Mild-to-Moderate Cubital Tunnel Syndrome: A Case–Control Study

**DOI:** 10.3390/healthcare13212764

**Published:** 2025-10-30

**Authors:** Michał Wieczorek, Tomasz Wolny

**Affiliations:** 1 Department of Theoretical and Practical Basics of Physical Therapy, The Jerzy Kukuczka Academy of Physical Education, Mikołowska 72A, 40-065 Katowice, Poland; t.wolny@awf.katowice.pl; 2 Institute of Physiotherapy and Health Sciences, The Jerzy Kukuczka Academy of Physical Education, Mikołowska 72A, 40-065 Katowice, Poland

**Keywords:** cubital tunnel syndrome, overall health status, entrapment syndrome, peripheral neuropathy, physiotherapy, physical health problems, mental health problems

## Abstract

**Objectives**: This cross-sectional case–control study, conducted at the Department of Physiotherapy, Academy of Physical Education, in Katowice, between July 2022 and April 2023, aimed to assess the overall health status (OHS) of patients with mild-to-moderate cubital tunnel syndrome (CuTS) compared with a healthy control population. **Methods**: The SF-36 questionnaire was used to evaluate the following domains: physical functioning (PF), role limitations due to physical health problems (RP), bodily pain (BP), general health (GH), vitality (VT), social functioning (SF), role limitations due to emotional problems (RE), and mental health (MH). **Results**: A total of 152 participants were enrolled in the study. The CuTS and control groups consisted of 82 and 70 individuals, respectively. The CuTS group had significantly lower scores in most domains when compared with the control group; however, no significant difference was observed in the BP domain. Notably, the VT domain showed a statistically significant difference in favor of the CuTS group, indicating slightly higher perceived vitality despite functional limitations. **Conclusions**: Mild and moderate forms of CuTS are associated with significant deterioration in general health, encompassing both physical and psychological components, as assessed by the SF-36 questionnaire; however, no significant difference was observed in the BP domain, suggesting that the burden of CuTS may manifest more strongly in functional, psychosocial, and mental health aspects rather than in generalized pain perception. These findings highlight the importance of a comprehensive evaluation and management strategy that addresses both physical and psychosocial dimensions of patient care.

## 1. Introduction

Cubital tunnel syndrome (CuTS) is the second most common entrapment neuropathy of the upper limb, after carpal tunnel syndrome (CTS) [1,2,3]. The condition is caused by chronic compression of the ulnar nerve at the elbow, most commonly in the region between the medial epicondyle and the olecranon. Its etiology may be mechanical (static or dynamic compression, overload, trauma) or anatomical (e.g., the presence of the anconeus epitrochlearis muscle) [2]. Clinically, CuTS presents with sensory and motor disturbances, including paresthesia in the fourth and fifth fingers, reduced grip strength, atrophy of intrinsic hand muscles, and pain that worsens with elbow flexion. Other potential compression sites include Struthers’ arch, Osborne’s ligament, the flexor carpi ulnaris aponeurosis, and the ulnar tunnel [2,3]. In mild-to-moderate CuTS, sensory symptoms predominate, often worsening at night or during prolonged elbow flexion (e.g., while talking on the phone). As neuropathy progresses, motor symptoms such as grip weakness, intrinsic muscle atrophy, and positive Froment and Wartenberg signs become evident [1,4,5].

The management of CuTS may involve conservative strategies (such as activity modification, splinting, physiotherapy, or electrophysical modalities) or surgical procedures [1,2,3,4,6,7,8]. Although various approaches have shown beneficial effects, existing evidence does not clearly indicate the superiority of any one method over another, and no universally accepted “gold standard” exists [1,2,3,4,6,7,8]. Importantly, from the patient’s perspective, the most meaningful outcomes are pain relief, functional recovery, and improved daily activities, which directly enhance overall health status (OHS) and quality of life.

When evaluating treatment outcomes in CuTS, objective measures, such as nerve conduction studies, cross-sectional area (CSA) assessment, discriminative sensation [9], and sensory threshold testing, are important [10]. Equally important, however, are patient-reported outcomes, which can be grouped into three main categories. First, pain assessment tools such as the Visual Analogue Scale (VAS), the Numeric Pain Rating Scale (NPRS), and the Leeds Assessment of Neuropathic Symptoms and Signs (LANSS) help to differentiate neuropathic from non-neuropathic pain [11,12,13]. Second, function-specific questionnaires—including the Global Rating of Change Scale (GROC), the QuickDASH, the Self-Administered Questionnaire for Ulnar Neuropathy at the Elbow (SQUNE), and the Patient-Specific Functional Scale (PSFS)—are used to evaluate limb function and patient-perceived activity limitations [14,15,16,17]. Finally, OHS, encompassing both physical and mental aspects, is most widely assessed using the SF-36 questionnaire [18,19,20,21,22,23,24]. The SF-36 was chosen in the present study because it is a validated and widely applied instrument that captures multiple dimensions of health, including both physical and psychological components [25]. Moreover, it has been extensively used in research on peripheral neuropathies and compression syndromes of the upper limb [26], ensuring the comparability of results and sensitivity in detecting health-related quality-of-life impairments [23].

This measure has been extensively studied in other peripheral neuropathies. For example, the SF-36 and the Boston Carpal Tunnel Questionnaire (BCTQ) have been repeatedly applied in the context of CTS to assess the condition’s impact on daily functioning [27,28,29]. Similarly, in diabetic neuropathy, worsening OHS has been shown to correlate with greater pain and functional impairment [30,31]. In brachial plexus injuries, OHS assessment is also an important component of treatment evaluation [32]. However, few studies have comprehensively assessed OHS in patients with CuTS using the SF-36.

Wolny et al. [26] evaluated the overall health status (OHS) in patients with mild-to-moderate carpal tunnel syndrome (CTS), compared with controls, using the SF-36 questionnaire. They reported significantly lower scores in physical domains (PF, RP, BP, and GH), whereas differences in mental domains were less pronounced and mainly related to vitality and social functioning. However, no previous studies have comprehensively assessed OHS in patients with CuTS using the SF-36 tool [18,27]. Existing research has focused primarily on electrophysiological or functional parameters [9,14,17], with limited attention to patients’ subjective perceptions of health and quality of life. The present study aims to fill this gap by providing a multidimensional evaluation of physical, emotional, and social aspects of well-being in CuTS and extends the evidence derived from studies on CTS and other upper limb neuropathies [27,30,33], contributing new insights into how CuTS affects both physical function and psychosocial health.

Little is known about the OHS of patients with mild-to-moderate CuTS. However, it is well established that CuTS can lead to functional impairments such as reduced grip strength, intrinsic hand muscle atrophy, and limitations in performing activities of daily living, which substantially affect patient independence [1,4,5]. CuTS may occur in up to 5.9% of the general population [4], and its impact on daily life can be significant. This study aimed to assess the OHS of patients with mild-to-moderate CuTS, compared to that of a control population, using the SF-36 questionnaire.

## 2. Materials and Methods

### 2.1. Study Design

**Figure 1:**
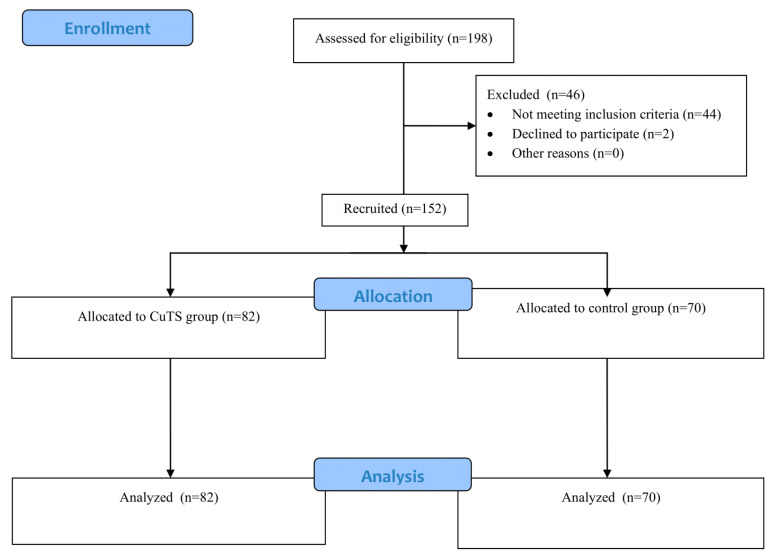
Flowchart.

This was a case–control study conducted in the Silesia region of Poland. Patients with CuTS were recruited from several medical clinics between 2022 and 2023 and included in the case group. A total of 198 individuals were assessed for eligibility; 46 were excluded (44 not meeting inclusion criteria and 2 declining to participate). The final study sample comprised 152 participants—82 patients with CuTS and 70 healthy controls. Data from the CuTS group were obtained during baseline assessments of a randomized controlled trial investigating the effects of physiotherapy on CuTS symptoms (ACTRN12621001623886). A flow diagram summarizing the recruitment and inclusion process is presented in Figure 1.

The study was conducted in accordance with the Declaration of Helsinki and approved by the local bioethics committee (Approval No. 8/2019). Written informed consent was obtained from all participants prior to study enrollment. The recruitment of participants started on 7 July 2022 and ended on 29 December 2022, and data collection was completed on 10 April 2023. Participants were informed about the purpose and procedures of the study and were assured of confidentiality and anonymity. All sensitive data were pseudonymized and stored on secure, password-protected institutional servers. Data handling followed the ethical standards of the Declaration of Helsinki and institutional regulations.

No formal sample size calculation was performed. The study was based on a convenience sample, including all consecutive patients with mild-to-moderate CuTS who met the inclusion criteria during the recruitment period, along with age- and sex-matched controls.

### 2.2. Participants

Participants with CuTS were included in the study if they met the following criteria:

- Subjective symptoms of peripheral ulnar neuropathy (pain, numbness, tingling in the innervation area of the ulnar nerve);
- Objective clinical signs (symptom exacerbation with elbow flexion and/or a positive elbow flexion test, positive Tinel’s sign at the elbow, McGowan grade I (mild sensory symptoms without motor weakness) or grade II (sensory symptoms accompanied by mild intrinsic muscle weakness));
- Sensory abnormalities (e.g., detected through tests assessing sensation in the innervation area of the ulnar nerve, such as two-point discrimination or sensory threshold testing);
- Ultrasound abnormalities (increased CSA and/or increased stiffness of the ulnar nerve);
- Nerve conduction disturbances (motor conduction velocity < 49.3 m/s in the ulnar nerve).

A diagnosis of CuTS was confirmed only if conduction disturbances were present in addition to at least two of the above clinical, sensory, or imaging abnormalities.

Controls were eligible if they were in good general health, provided informed consent to participate in the study, and reported no symptoms suggestive of upper limb neuropathy.

Exclusion criteria for both the CuTS and control groups included the following: prior surgery, current use of steroid or nonsteroidal medications, diagnosed cervical radiculopathy, tendinitis, rheumatic diseases, diabetes mellitus, pregnancy, a history of elbow trauma, or evidence of muscle atrophy in the hypothenar region.

### 2.3. Measurements

OHS in both groups was assessed using the SF-36 questionnaire [25]. This is a self-report tool comprising 11 questions with a total of 36 statements, allowing for analysis of eight aspects of general health. The following areas are considered: physical functioning (PF), role limitations because of physical health problems (RF), bodily pain (BP), general health (GH), vitality (VT), social functioning (SF), role limitations because of emotional problems (RE), and mental health (MH). Each domain is scored from 0 to 100. The average score of a sub-area was calculated by adding up the scores indicated by the participant and dividing by the number of items in the component. In this way, values for all eight OHS components were obtained.

The Physical Component Summary (PCS) and Mental Component Summary (MCS) scores were calculated according to the standard weighted algorithm described by Ware and Gandek (1998) [25]. Each SF-36 domain was standardized (z-score transformation) and multiplied by its respective factor scoring coefficient before summation. This method accounts for the differential contribution of each domain to physical and mental health, ensuring comparability with studies using the norm-based scoring approach [25].

### 2.4. Statistical Analysis

A *t*-test for independent samples was used to calculate mean values, standard deviations (SDs), and confidence intervals. To test the main assumptions of the *t*-test, Levene’s test (equality of variance) and the Shapiro–Wilk test (normal distribution) were performed, and their results are reported in the Section 3. The chi-square test was used to assess the homogeneity of categorical demographic variables between groups, specifically sex distribution. Age was treated as a continuous variable and analyzed using the *t*-test. A *p*-value < 0.05 was considered statistically significant.

## 3. Results

A total of 152 participants were enrolled in the study. The CuTS group consisted of 82 individuals (54 men, 28 women), while the control group included 70 individuals (50 men, 20 women). Detailed results are presented in Table 1.

There were no significant differences between groups in terms of sex distribution (χ^2^ = 0.34, *p* = 0.56) or age (*t* = 0.29, *p* = 0.77).

Preliminary analyses confirmed that assumptions of normality (Shapiro–Wilk test, all *p* > 0.05) and homogeneity of variance (Levene’s test, all *p* > 0.05) were satisfied. Therefore, between-group differences in the SF-36 domains were assessed using Student’s *t*-test.

The experimental group underwent detailed clinical, electrophysiological, ultrasound, and subjective pain assessments.

Clinical severity was evaluated using the McGowan scale, with a mean score of 1.49 ± 0.50 (range: I–II°), confirming mild-to-moderate functional impairment.

Motor conduction velocity (MCV) testing revealed a mean value of 35.82 ± 5.49 m/s (range: 25.30–47.40), indicating moderate slowing of ulnar nerve conduction.

Ultrasonographic assessment showed a mean ulnar nerve cross-sectional area (CSA) of 11.06 ± 1.58 mm^2^ (range: 7.77–14.21 mm^2^). Shear wave elastography (SWE) was also performed, with a mean nerve stiffness of 30.45 kPa ± 4.70 (range: 19.66 to 39.10), which may be indicative of structural changes within the nerve [33,34].

Pain intensity was evaluated separately for day and night using the Numerical Pain Rating Scale (NPRS). The mean nocturnal pain score was 4.71 ± 1.04 (range: 3–7), while the mean daytime pain score was 2.60 ± 0.74 (range: 1–4), indicating predominantly nocturnal symptoms.

No CuTS symptoms or functional impairments were observed in the control group. There were also no functional problems.

### 36-Item Short-Form Health Survey

OHS was assessed with the SF-36 questionnaire. The CuTS group had significantly lower scores in most domains compared with the control group, except the bodily pain (BP) domain, where no significant difference was observed. Notably, the vitality (VT) domain showed a statistically significant difference in favor of the CuTS group. Detailed results are presented in Table 2, Table 3 and Table 4.

**Figure 2:**
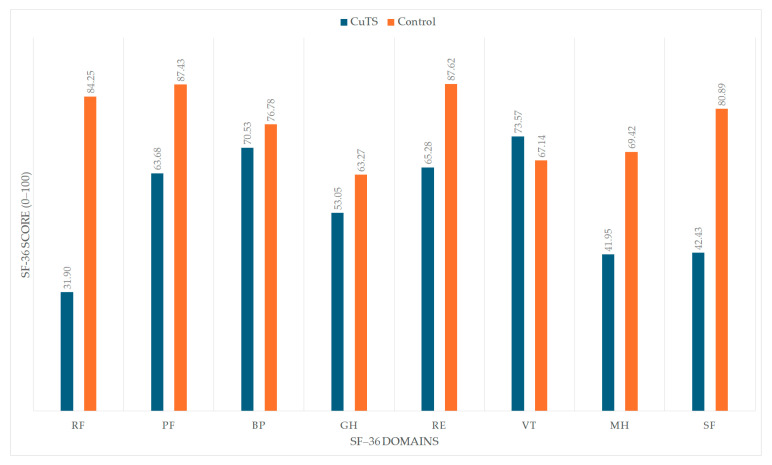
Comparison of SF-36 domain scores between patients with cubital tunnel syndrome (CuTS) and healthy controls. Bars represent mean values (0–100 scale). PF—physical functioning; RF—role physical; BP—bodily pain; GH—general health; RE—role emotional; VT—vitality; MH—mental health; SF—social functioning. Higher scores indicate better health-related quality of life.

Figure 2 visually illustrates the differences in SF-36 domain scores between patients with CuTS and healthy controls. The CuTS group demonstrated significantly lower scores across most domains, including PF, RF, GH, RE, SF, and MH, while no significant difference was observed in BP. Interestingly, the VT domain showed slightly higher scores in the CuTS group, possibly reflecting adaptive coping despite disease-related limitations.

## 4. Discussion

This study demonstrated that individuals with mild-to-moderate CuTS scored significantly lower on most SF-36 domains compared with controls. The greatest impairments were found in the PF, RP, GH, RE, SF, and MH domains (all *p* < 0.001). The VT domain showed slightly higher scores in the CuTS group (*p* = 0.036), while no significant difference was observed in the BP domain (*p* = 0.209). Overall, these findings confirm that CuTS substantially impairs both physical and psychosocial dimensions of overall health, with functional and emotional limitations having the strongest impact.

The present study provides specific insight into the OHS of patients with CuTS. The results clearly demonstrate that CuTS significantly impairs both physical and psychosocial components of health, as evidenced by markedly lower SF-36 scores in PF, RP, GH, RE, SF, and MH domains. Even mild-to-moderate CuTS can have a measurable impact on quality of life, primarily due to hand dysfunction, restricted participation in daily activities, and emotional stress related to chronic symptoms. Previous reports concerning other entrapment neuropathies, such as CTS, have revealed similar reductions in SF-36 domains [26]; however, the current results extend this evidence to ulnar nerve entrapment, highlighting that CuTS presents its own distinctive pattern of functional and psychosocial burden. Similar findings were reported by Reischl and MacDermid [35], who demonstrated that patients with ulnar neuropathy showed lower mental health scores compared to those with distal radius fractures, suggesting that psychosocial disturbances may play a more dominant role in nerve compression than in post-traumatic conditions.

Comparable reductions in health-related quality of life have also been reported in various neuropathies, including neurogenic thoracic outlet syndrome (NTOS), Charcot–Marie–Tooth disease (CMT1A), peripheral nerve injury (PNI), and other chronic immune-mediated neuropathies such as anti-MAG neuropathy, systemic lupus erythematosus, and primary Sjögren’s syndrome [36,37,38,39,40,41,42]. Across these conditions, chronic pain, sensory disturbances, and functional limitations have been shown to markedly decrease SF-36 scores in both physical and mental domains. These findings support the present results and indicate that compression neuropathies, irrespective of anatomical site, produce widespread systemic and psychosocial consequences beyond local nerve dysfunction. Together, this evidence suggests that functional limitations and pain may have a greater influence on overall health status than objective electrophysiological indicators, confirming that CuTS exerts a comparable dual burden of physical and psychosocial impairment.

Consistent with the present results, Meyer-Rosberg et al. [43] demonstrated that neuropathic pain and upper limb nerve compression disorders substantially reduce health-related quality of life, particularly within physical and mental domains.

Moreover, Guilfoyle et al. [44] discussed possible “ceiling” and “floor” effects in SF-36 scoring that could limit sensitivity in certain domains. The absence of such effects in our dataset reinforces the robustness and reliability of the SF-36 in assessing OHS among CuTS patients, as it effectively detected both significant impairments and compensatory psychosocial adaptations without skewing toward extreme score values.

Rafiq et al. [45] emphasized that the physical components of quality of life are the most affected in patients with peripheral nerve injuries, consistent with our findings showing significant reductions in PF, RP, and GH domains among CuTS patients. Vickrey et al. [46] demonstrated that mental health outcomes are closely intertwined with functional impairments, supporting our observation that reduced upper limb function in CuTS corresponds with lower RE, SF, and MH scores. Thomsen et al. [47] further noted that the chronic nature of neuropathic symptoms contributes to long-term psychosocial adaptation, aligning with the elevated VT scores seen in our CuTS cohort. This counterintuitive result may reflect adaptive psychological mechanisms such as cognitive reframing, acceptance, and reorganization of daily activities, which help affected individuals to maintain a subjective sense of vitality despite ongoing physical limitations. Similar adaptive responses have been described in other peripheral neuropathies and musculoskeletal conditions, where persistent symptoms prompt patients to adjust their expectations and coping strategies, preserving or even enhancing perceived well-being [46,47,48]. Similarly, Çağlar et al. [48] reported that even mild compression neuropathies can alter self-perceived health and daily functioning, reinforcing our conclusion that early-stage CuTS already imposes measurable limitations on both physical and emotional well-being.

Night pain is one of the most characteristic and disabling symptoms of CuTS, often resulting from increased intraneural pressure during elbow flexion in sleep [49,50,51]. This mechanism likely contributes to reduced PF and GH scores on the SF-36, and should be addressed through positioning strategies and elbow extension splints to improve patient-reported outcomes.

An interesting observation was the lack of a significant difference between groups in the BP domain of the SF-36 despite clearly reported pain. Similar findings were reported by MacDermid et al. [52], indicating that the BP subscale of the SF-36 may have limited sensitivity in detecting pain localized to the upper limb compared with ulnar neuropathy-specific tools such as the PRUNE. In our cohort, pain was clearly reflected in the NPRS scores, particularly nocturnal pain (mean 4.71 ± 1.04), confirming the presence of clinically relevant pain despite the absence of significant differences in the SF-36 BP domain. This suggests that the pain burden in CuTS may manifest more strongly through functional and psychosocial limitations—such as role restrictions, social functioning, and mental health—rather than through generalized pain perception assessed by the BP scale.

Previous studies have shown that pain intensity is a key determinant of health-related quality of life in peripheral neuropathies, influencing both physical and psychological outcomes [53,54]. Moreover, certain CuTS symptoms are neuropathic in nature—such as burning, hypersensitivity, or paroxysmal pain—which supports the use of screening tools like DN4, S-LANSS, or PainDETECT in future research to better differentiate neuropathic from non-neuropathic pain [55,56,57]. Implementing such tools could allow for more precise mapping of pain types to specific SF-36 domains and guide more targeted therapeutic interventions.

## 5. Clinical Implications

A comprehensive approach to CuTS management should integrate both physical and psychosocial perspectives. The SF-36 questionnaire can serve not only as a global outcome measure, but also as a practical tool to guide individualized rehabilitation. Low PF and RP scores may indicate the need for focused physical therapy aimed at improving upper limb strength, range of motion, and ergonomic function, whereas reductions in the MH, RE, or SF domains emphasize the importance of addressing emotional distress, coping, and social participation. Regular reassessment with the SF-36 enables clinicians to track progress in both functional and mental domains, ensuring a balanced, patient-centered rehabilitation strategy. Combining generic tools such as the SF-36 with CuTS-specific assessments (e.g., PRUNE, NPRS, or DN4) may further enhance clinical decision-making and provide a more comprehensive picture of recovery and quality of life.

## 6. Limitations of the Study

Our study has some limitations. It was conducted exclusively in the Silesian region of Poland, which may limit the generalizability of the findings to the entire national population. Demographic differences, variations in healthcare access, and cultural preference factors across regions may influence OHS outcomes. Because patients with CuTS were recruited from outpatient rehabilitation and neurology clinics, a potential selection bias cannot be excluded. These individuals may represent patients with more pronounced symptoms or higher health awareness compared with the general CuTS population. The health status of control participants was verified through clinical interviews and self-reporting to ensure the absence of upper limb neuropathic symptoms, musculoskeletal disorders, or other chronic conditions that could affect overall health perception. Furthermore, the cross-sectional design does not permit analysis of longitudinal changes in PCS and MCS or assessment of treatment effects over time. Future multicenter, prospective studies covering diverse regions and incorporating long-term follow-up are needed to provide stronger evidence.

Additionally, although participants were screened to exclude major medical conditions, the potential influence of unreported or subclinical comorbidities cannot be entirely ruled out. Minor musculoskeletal disorders, mood disturbances, or other psychological factors may have affected participants’ self-perceived health and consequently influenced SF-36 scores. Future studies should include more detailed assessments to control for these potential confounders.

Furthermore, no formal a priori sample size calculation was performed. The study was based on a convenience sample of all eligible participants recruited during the study period, which may limit the precision of effect estimation. However, a post hoc power analysis based on the observed effect sizes for the SF-36 summary measures (PCS: d = 0.77; MCS: d = 1.06) indicated high statistical power (1–β = 0.93 and 0.99, respectively, at α = 0.05), suggesting that the sample of 152 participants was adequate to detect clinically meaningful differences.

## 7. Conclusions

Mild and moderate forms of CuTS are associated with significant deterioration in general health, encompassing both physical and psychological components, as assessed by the SF-36 questionnaire. The largest deficits were observed in the domains of role limitations, physical functioning, mental health, and social functioning. However, no significant differences were found in the bodily pain domain, suggesting that the negative impact of CuTS on overall health may be driven more by functional and psychosocial limitations than by generalized pain perception. These findings highlight the importance of a comprehensive evaluation and management strategy that addresses both the physical and psychosocial aspects associated with this condition. Incorporating multidimensional assessment tools such as the SF-36 into routine clinical practice can enhance rehabilitation planning and improve treatment outcomes.

In clinical follow-up, particular attention should be paid to the SF-36 domains that showed the most pronounced impairments in this study—namely, physical functioning (PF), role limitations due to physical problems (RP), social functioning (SF), role limitations due to emotional problems (RE), and mental health (MH). Monitoring these domains may help clinicians to track recovery, evaluate the effectiveness of therapy, and better tailor rehabilitation programs to address both physical performance and psychosocial well-being.

## Figures and Tables

**Table 1:** Basic characteristics: mean value (standard deviations) and mean differences (95%CI).

Characteristics		Group		
	CuTS (n = 82)	Control (n = 70)	CuTS Minus Control (95%CI)	*p*-Value
Age (year); (mean, SD)	53.27 (7.20)	51.83 (8.24)	1.44 (−3.74 to 1.47)	*p* = 0.238
Body mass (kg); (mean, SD)	75.41 (12.71)	75.34 (8.07)	0.07(−4.34 to 4.49)	*p* = 0.411
Body height (cm); (mean, SD)	173.69 (10.37)	171.47 (14.86)	2.22 (−0.97 to 5.42)	*p* = 0.177
BMI (mean, SD)	25.11 (3.40)	25.91 (3.40)	−0.8 (−1.88 to 0.29)	*p* = 0.991
Sex distribution (F/M %)	34/66%	29/71%	-	*p* = 0.461 ^a^

Abbreviations: CuTS—cubital tunnel syndrome; BMI—body mass index; n—number of people; %—percentage value; SD—standard deviation; 95% CI = 95% confidence interval; ^a^—chi-square test for sex distribution; F/M %—proportion of females to males in percentage.

**Table 2:** SF-36 questionnaire (physical components)—mean value (standard deviations) and mean differences (95%CI).

SF-36 (Physical Components)	Group			
	CuTS (n = 82) (Mean ± SD)	Control (n = 70) (Mean ± SD)	CuTS Minus Control (95%CI)	*p*-Value
RF	31.90 ± 30.90	84.25 ± 29.93	−52.35 (−62.01 to −42.68)	**
PF	63.68 ± 22.13	87.43 ± 14.86	−23.75 (−29.85 to −17.64)	**
BP	70.53 ± 36.74	76.78 ± 21.34	−6.25 (−16.03 to 3.53)	*p* = 0.209
GH	53.05 ± 13.60	63.27 ± 17.45	−10.22 (−15.11 to −5.32)	**

Abbreviations: SF-36—36-Item Short-Form Health Survey; CuTS—cubital tunnel syndrome; RF—role limitations because of physical health problems; PF—physical functioning; BP—bodily pain; GH—general health; SD—standard deviation; 95% CI = 95% confidence interval; **—*p* < 0.001 indicates statistical significance.

**Table 3:** SF-36 questionnaire (mental components)—mean value (standard deviations) and mean differences (95%CI).

SF-36 (Mental Components)	Group			
	CuTS (n = 82) (Mean ± SD)	Control (n = 70) (Mean ± SD)	CuTS Minus Control (95%CI)	*p*-Value
RE	62.28 ± 16.64	87.62 ± 27.32	−25.34 (−29.33 to 15.35)	**
VT	73.57 ± 20.20	67.14 ± 17.31	6.43 (0.41 to −12.44)	*
MH	42.11 ± 12.77	69.42 ± 16.40	−27.31 (−32.06 to −22.86)	**
SF	42.43 ± 13.93	80.89 ± 19.12	−38.46 (−43.68 to −33.25)	**

Abbreviations: SF-36—36-Item Short-Form Health Survey; CuTS—cubital tunnel syndrome; RE—role limitations because of emotional problems; VT—vitality; MH—mental health; SF—social functioning; SD—standard deviation; 95% CI = 95% confidence interval; *—*p* < 0.01 and **—*p* < 0.001 indicates statistical significance.

**Table 4:** SF-36 questionnaire (PCS and MCS)—mean value (standard deviations) and mean differences (95% CI).

SF-36 (Summary)	Group			
	CuTS (n = 82) (Mean ± SD)	Control (n = 70) (Mean ± SD)	CuTS Minus Control (95%CI)	*p*-Value
PCS	43.32 ±9.80	50.46 ±7.40	−7.14(−9.80 to −4.48)	**
MCS	39.86 ±9.30	49.36 ± 8.10	−9.51 (−12.30 to –6.72)	**

Abbreviations: SF-36—36-Item Short-Form Health Survey; CuTS—cubital tunnel syndrome; PCS—physical component summary; MCS—mental component summary; SD—standard deviation; 95% CI = 95% confidence interval; **—*p* < 0.001 indicates statistical significance. PCS and MCS were computed using the standard SF-36 v1 norm-based scoring algorithm with factor score coefficients from a normative population, as described in the SF-36 manual [25], and expressed as T-scores (mean = 50, SD = 10).

## Data Availability

The data presented in this study are available on request from the corresponding author due to specify the reason for the restriction.

## Abbreviations

The following abbreviations are used in this manuscript:

anti-MAG neuropathy	Antymyelin-associated glycoprotein neuropathy
Q-DASH	Quick Disabilities of the Arm, Shoulder, and Hand
SQUNE	Self-Administered Questionnaire for Ulnar Neuropathy at the Elbow
LANSS	Leeds Assessment of Neuropathic Symptoms and Signs
CMT1A	Charcot–Marie–Tooth disease
PRUNE	Patient-Rated Ulnar Nerve Evaluation
PSFS	Patient-Specific Functional Scale
CuTS	Cubital tunnel syndrome
NPRS	Numeric Pain Rating Scale
GROC	Global Rating of Change
BCTQ	Boston Carpal Tunnel Questionnaire
NTOS	Neurogenic thoracic outlet syndrome
QLIP	Quality of Life Impairment by Pain
SF-36	36-Item Short-Form Health Survey
SF-12	12-Item Short-Form Health Survey
DN-4	Douleur Neuropathique 4
PNI	Peripheral nerve injury
UNE	Ulnar neuropathy
DRF	Distal radius fracture
CSA	Cross-sectional area
NCS	Nerve conduction study
BMI	Body mass index
CTS	Carpal tunnel syndrome
VAS	Visual Analogue Scale
PCS	Physical Component Summary
MCS	Mental Component Summary
MCV	Motor conduction velocity
SWE	Shear wave elastography
PF	Physical functioning
RF	Role limitations because of physical health problems
BP	Bodily pain
GH	General health
VT	Vitality
SF	Social functioning
RE	Role limitations because of emotional problems
MH	Mental health
CI	Confidence interval
SD	Standard deviation
